# MLIP: using multiple processors to compute the posterior probability of linkage

**DOI:** 10.1186/1471-2105-9-S6-S2

**Published:** 2008-05-28

**Authors:** Manika Govil, Alberto M Segre, Veronica J Vieland

**Affiliations:** 1 Department of Oral Biology and Center for Craniofacial and Dental Genetics, School of Dental Medicine, University of Pittsburgh, Pittsburgh, Pennsylvania, USA; 2 Department of Computer Science, The University of Iowa, Iowa City, Iowa, USA; 3 Battelle Center for Mathematical Medicine, The Research Institute at Nationwide Children's Hospital and The Ohio State University, Columbus, Ohio, USA

## Abstract

**Background:**

Localization of complex traits by genetic linkage analysis may involve exploration of a vast multidimensional parameter space. The posterior probability of linkage (PPL), a class of statistics for complex trait genetic mapping in humans, is designed to model the trait model complexity represented by the multidimensional parameter space in a mathematically rigorous fashion. However, the method requires the evaluation of integrals with no functional form, making it difficult to compute, and thus further test, develop and apply. This paper describes MLIP, a multiprocessor two-point genetic linkage analysis system that supports statistical calculations, such as the PPL, based on the full parameter space implicit in the linkage likelihood.

**Results:**

The fundamental question we address here is whether the use of additional processors effectively reduces total computation time for a PPL calculation. We use a variety of data – both simulated and real – to explore the question "how close can we get?" to linear speedup. Empirical results of our study show that MLIP does significantly speed up two-point log-likelihood ratio calculations over a grid space of model parameters.

**Conclusion:**

Observed performance of the program is dependent on characteristics of the data including granularity of the parameter grid space being explored and pedigree size and structure. While work continues to further optimize performance, the current version of the program can already be used to efficiently compute the PPL. Thanks to MLIP, full multidimensional genome scans are now routinely being completed at our centers with runtimes on the order of days, not months or years.

## Background

From at least as far back as 1866, when Gregor Mendel published his findings on the laws of inheritance based on experiments with pea plants, statistics has been used as an important tool for genetic studies of inherited traits. With advances in technology and the development of increasingly complex models for analysis, the use of computer science has become integral for all such statistical genetic studies.

One research area of interest for geneticists is locating on the genome the gene(s) responsible for inherited traits. This localization is facilitated by linkage analysis, a statistical genetic method. The method relies on violations of Mendel's second law of inheritance, which states that genes assort independently, to estimate the proximity of trait genes to known genomic locations (genetic markers). The model-based linkage statistic is the LOD score, defined as the log_10 _ratio of the likelihood of the observed data, divided by the likelihood assuming "no linkage." This statistic utilizes genetic map, pedigree, and trait model information (*e.g.*, the population frequency of the trait, and the *penetrance *or probability that an individual with a given genetic combination at the trait locus manifests the trait) to obtain an estimate of the recombination fraction *θ*, the traditional measure of genetic distance between two genomic locations.

A typical linkage analysis therefore involves computing likelihoods in several parameters in order to summarize the information regarding *θ*. However, complicating factors exist. While sex-averaged genetic maps are normally used in analyses, the true male-female maps are of different lengths. This use of sex-averaged maps results in map misspecification. Heterogeneity among pedigrees from different populations is another complicating factor. Finally, the trait may be complex, possessing a genetic component but not a simple mode of inheritance. For such traits, linkage analysis may involve exploration of a vast multidimensional parameter space.

The posterior probability of linkage, or PPL, [1,2] is a class of linkage statistics designed to model both sex-specific maps and the trait model complexity represented by the multidimensional parameter space in a mathematically rigorous fashion. It also accommodates both inter- and intra-population heterogeneity by using Bayesian sequential updating over data subsets. However, the method requires the evaluation of integrals with no functional form, making it difficult to compute, and thus further test, develop and apply.

This paper describes MLIP (*Multiprocessor LInkage analysis Program*), a new, dual-language system that calculates, in parallel, two-point LOD scores by pedigree and marker over a user-specified range of genetic parameters and male and female recombination fractions. MLIP is designed to facilitate genetic linkage analysis (gene mapping) by allowing the coverage of a complex, multidimensional parameter space using partitioning and parallelization of the computation. Motivation for the development of MLIP derives from the utility of representing the underlying linkage likelihood explicitly as a function of all implicit parameters to allow computation of the PPL.

MLIP utilizes NICE, the *Network Infrastructure for Combinatorial Exploration *[3], a network computing infrastructure for dynamic parallelization across a grid of processors. While parallelization has been considered previously in connection with linkage analysis (*e.g.*, see [4-10]), none of these approaches have dealt with computation over the trait parameter space, the focus of the present work.

## Methods

The simplest characterization of MLIP is as a two-layer model: an *inner layer *that is used to compute each individual LOD score, and an *outer layer *that oversees the systematic exploration of the multidimensional parameter space by dividing it into chunks, managing the distribution of these chunks to other processors, collecting the results, and subsequently writing them to disk.

**Figure 1:**
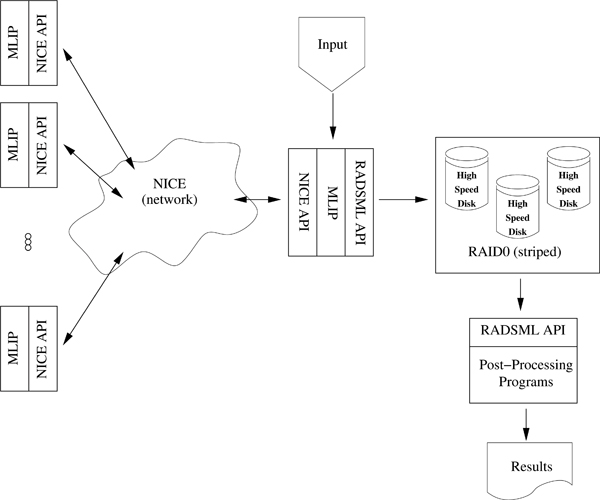
**MLIP: Overview**. MLIP relies on NICE to provide a dynamic hierarchy of processors, all of which obtain portions of the parameter space from their parent or root node. These processors in turn, return results to the root node for storage on disk. MLIP invokes functions provided by RADSML (*Random Access Data Storage for MLip*) to store the output compactly in a special file.

Figure 1, provides an overview of MLIP and the different internal and external components that comprise the distributed computing package. The output of MLIP, essentially a large set of single-precision LOD scores describing the likelihood surface over a multidimensional parameter space, can then be used to calculate the PPL integral.

### Inner layer: computing the LOD scores

The two-point PPL is by definition the definite integral over the interval [0, 0.5) of the recombination fraction (*θ*). In other words, it is the probability that *θ *< 1/2 given the data. For marker data *M*, and trait data *T*, the two-point PPL can be expressed in terms of LOD scores as [1,11]:

(1) $$PPL=\frac{P(L)\int_{\theta \in \left[0,\frac{1}{2}\right)}BR(\theta )f\left(\theta |L\right)d\theta }{P(L)\int_{\theta \in \left[0,\frac{1}{2}\right)}BR(\theta )f\left(\theta |L\right)d\theta +\left(1-P\left(L\right)\right)}$$

Where:

(2) $$BR\left(\theta \right)=\int_{\alpha }\int_{g}10^{HLOD(\theta ,\alpha ,g)}f\left(\alpha \right)f\left(g\right)d\alpha \;dg$$

In these equations, *L *indicates linkage, ***g ***is a vector of trait parameters, *θ *is the recombination fraction, *α *is the admixture parameter, and *f*(***g***), *f*(*θ*), *f*(*α*) are prior distributions for ***g***, *θ*, and *α *respectively. BR(*θ*), a function of *θ*, is the direct analogue of the usual likelihood ratio (LR). Since it is also proportional to the posterior density of *θ*, the BR is simply multiplied across the data (sub)sets to sequentially update the PPL over multiple sets of data and the PPL is recomputed using Eq. 2 [12]. Nuisance parameters are integrated out of the BR independently for each set of data, thereby allowing these parameters to vary independently across data sets, while evidence regarding *θ *is accumulated across data sets. Since the integrand for computing the PPL involves several integrals and has no analytical functional form, it is approximated by averaging heterogeneity LOD scores (HLODs) over a discretized parameter space [13-15].

The inner layer of MLIP allowing computation of the LOD scores necessary for the PPL is LIPED [16], Jurg Ott's FORTRAN implementation of the Elston-Stewart algorithm [17]. LIPED has been selected as the inner layer of MLIP for several reasons. Since the program is an implementation of the Elston-Stewart algorithm, it is capable of handling the medium to large pedigrees regularly encountered in our own clinical collaborations.

Not only is LIPED both trusted and well tested, with the source code freely available, but it also has additional important advantages over some of the other existing Elston-Stewart implementations. Computing the PPL requires the flexibility to include a variety of genetic parameters in the model. LIPED supports sex-specific recombination fractions; accepts haplotype frequencies in a manner that allows modeling linkage with disequilibrium correctly; handles quantitative traits; models delayed onset; and supports *loops*, or complex pedigree structures where more than one path may exist between two individuals.

While the code is not new, when compared to more recent Elston-Stewart implementations (such as FASTLINK [18]), optimized to reduce the cost of a single LOD score calculation, somewhat surprisingly, the performance of the original LIPED code is still quite competitive overall. For some pedigree structures, it even provides better performance than more recent implementations. When tightly integrated into MLIP and with just a few small improvements, our modified LIPED core's performance is quite respectable (especially when compared to, e.g., using remote procedure calls to invoke multiple copies of FASTLINK over a network).

Finally, although LIPED is written in FORTRAN, the code is relatively compact and well documented. Since the modifications required to the original LIPED code did not involve extensive changes to its algorithmic core, our resulting calculations should inspire the same faith and confidence as those produced by the original LIPED code.

### Outer layer: partitioning over a cluster

Wrapped around LIPED is a new outer layer, coded in ANSI C. This layer is responsible for reading in the problem specification (pedigrees, markers, loop breakers, and the parameter grid) and checking it for errors; performing simple data transformations in the interests of efficiency (*e.g.*, *allele downcoding*, where the total number of alleles at each marker is adjusted down to the observed number); and breaking the space into appropriate size chunks to assign chunks to itself and other processors. It is also responsible for invoking LIPED and collecting computed LOD scores and storing them on disk. Finally, the layer ensures that the entire space is exhausted while taking appropriate measures to allow effective restarting of a previously interrupted computation via checkpointing.

**Figure 2:**
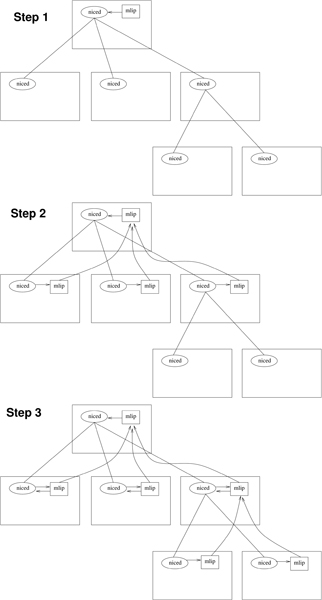
**The NICE hierarchy**. Propagation of parallel application down the NICE hierarchy. An application started on the root processor will spawn copies of the application recursively on descendent nodes.

MLIP utilizes the NICE infrastructure for distributed computing. Unlike peer-to-peer infrastructures, NICE models the distributed computing resource as a hierarchy of processors (see Figure 2). A NICE-enabled application communicates with the infrastructure via a set of callable functions contained in the NICE applications programmer's interface, or API. This linkable library contains functions that, when invoked, *e.g.*, request that new copies of the application be spawned on the same or other machines. It also contains functions to support communication between these multiple copies of the application once they are established. Note that the library does not actually contain code specific to any application, but rather only the handful of functions that are needed to efficiently parallelize appropriately designed serial applications.

Once the NICE infrastructure has spawned the appropriate configuration of processes over the nodes in the cluster, parallelization by partitioning involves having an idle slave processor ask its master for a chunk of work and then returning the results once the calculation is completed. Since a master processor may have multiple slaves, it should be able to partition independent chunks of its own assigned work among them. The model extends recursively, with each slave potentially having slaves of its own, subdividing its own chunk(s) into still smaller ones.

There are several complicating factors. First, this is a mixed-language system (LIPED is coded in FORTRAN), which requires special handling [19]. Second, the outer layer is responsible for managing the distribution of chunks to other processors. This requires care to keep all processors working at maximum capacity. A good partitioning scheme performs *load balancing *(ensures all available processors are kept busy) and is also *fault tolerant *(robust to processor or network failure). Good load balancing in heterogeneous computing environments presents a challenge. Differences in processor speed or memory capacity, and thus intrinsic computation speeds, makes it difficult to distinguish an unduly slow slave from one that has failed. Furthermore, for hierarchically-organized processors, some processors will appear much faster than others by virtue of the processor(s) available beneath them. Finally, in the case of MLIP, we have no *a priori *knowledge of the amount of CPU time required to perform a unit of work. This is due to the fact that not all LOD scores are created equal: differences in markers, pedigree size and structure, and pattern of unknown genetic marker and/or trait data, all affect the cost of the calculation.

Since load balancing and fault tolerance are intertwined, the load balancing scheme should also handle lost processors in a systematic fashion, "recovering" and reassigning inchoate chunks that had been assigned to slower or unresponsive processors. In this manner, faster processors cover more and more of a slower processor's assigned territory. If a processor has failed, the faster processors will eventually complete its work. If a processor is truly slow and completes its work before the faster processors report back, the redundantly assigned processors can be aborted without prejudice. This strategy implies that some LOD scores may be computed more than once to keep all CPUs busy and preserve fault tolerance. The key issue here is to tune the partitioning criteria such that all chunks are finished at approximately the same time and little redundant calculation actually occurs.

Finally, since the outer layer is ultimately responsible for collecting the LOD scores and efficiently streaming them to disk, it must keep up with the high rate with which results are generated by multiple processors. For MLIP, the protocol invoked when a slave completes a portion of its work is the most expensive and the number of LOD scores returned to the root can be quite large. For example, for 50 pedigrees, a typical genome-wide analysis with 400 markers over the regular space for the sex-specific PPL [15] entails the calculation, communication, and storage of 2.7 × 10^10 ^LOD scores, roughly 100 GB of output. This presents a special challenge for distributed computing. Typically, distributed applications will require that the input data sets, which can be quite large, be broadcast to individual processing elements at initialization, and that the subsequent results, which are typically small, be collected over the course of the calculation. Here, the input data sets, while not insignificant, are trivially small in comparison to the size of the results, which must be transmitted over the network for storage on disk, so that, *e.g.*, the PPL can be computed and recomputed with arbitrary priors.

To minimize communication costs, each slave node, regardless of its relative position in the hierarchy, communicates its results directly back to the root node, circumventing its own master.

## Results and discussion

The fundamental question addressed here is whether additional processors effectively reduce total computation time. While the use of N processors should, in theory, allow completion of the same calculation N times faster, few, if any, parallel computing efforts attain *linear speedup *in practice. This is because some of the work does not parallelize. For MLIP, all disk-writing costs are incurred solely by the root processor, while network communications are a necessary expense of all parallel systems.

**Figure 3:**
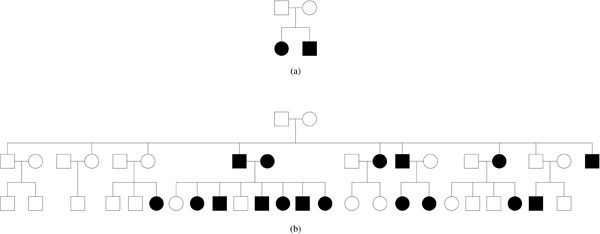
**Sample pedigrees for simulated data**. Circles represent females, squares represent males. Individuals with filled symbols manifest the trait under study. (a) An affected sib pair pedigree. Test sets A and B comprise 500 copies each of this pedigree with known (for set A) and unknown (set B) parental marker genotypes. (b) A multigenerational pedigree with 43 individuals. Test set C consists of 32 copies of this pedigree with all known marker genotypes. Set D consist of 4 copies of the pedigree with unknown marker genotypes for all but the last generation.

A sequence of empirical tests is used to explore the question "how close can we get?" to linear speedup. Since it is well known that pedigree size and structure, presence/absence of marker genotypic information, and number of marker alleles all affect the degree of difficulty and expected computation time for the Elston-Stewart algorithm, four simulated test sets have been generated from very different parts of the spectrum. We also test performance on a real data set later. To avoid confounding factors due to complex interactions between data features and computation time, the simulated sets are homogeneous, each consisting of many replicates or variants of one of two pedigree structures (see Figure 3).

Each test set has 32 markers. The markers for sets A and B have 2 alleles each, while for sets C and D the markers each have 16 alleles. The number of pedigree copies and marker alleles in each set reflects the expected complexity and computation time for the configuration. Sib pairs with two-allele markers and fully-known parental genotypes (set A) are expected to require the least amount of computation per LOD score, while larger pedigrees with more diverse markers and missing genotype information represent the other extreme (set D). Since the sets have different intrinsic difficulty, a different number of model parameters (the *grid size*) are selected for each set allowing a single processor trial to take approximately the same amount of time across sets. To examine the behavior of the system as the number of processors increases, each set is tested with n = 1, 2, 4, 8, 16, and 32 processors arranged in a uniform NICE hierarchy, where each processor has at most three child processors. The grid size is increased proportionally as n increases. Table 1 gives the minimum (for 1 processor) and maximum (for 32 processors) grid size for each test set.

**Table 1:** Grid size variation

**Processors**	**Set A**	**Set B**	**Set C**	**Set D**
1 CPU	33 × 10^6^	16 × 10^6^	10 × 10^5^	4096
32 CPUs	52 × 10^7^	26 × 10^7^	34 × 10^6^	13 × 10^4^

### Speedup and scalability

**Figure 4:**
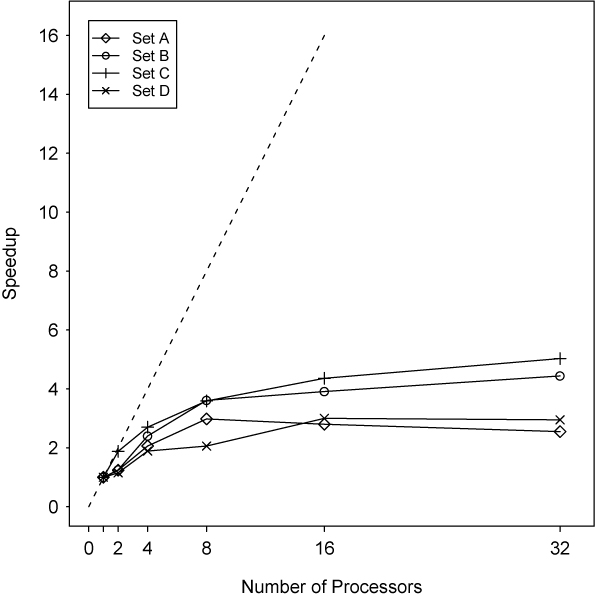
**Speedup by number of processors**. The dotted line represents linear speedup, which is difficult to achieve in practice.

Figure 4 plots the effective *speedup *obtained as the number of processors increases, where *speedup*, S_n_, is defined here as the ratio of the LODs/second on n processors to the LODs/second on a single processor: thus S_n _= n represents linear speedup.

Clearly, as seen from the plot, linear speedup is not being achieved for each of the test sets. What is interesting here is that the observed performance appears to be clustering by set, with B, C performing better than A, D.

To understand the reason behind this observed performance, we need to take a closer look at computation, disk-writing, and network costs of the root processor. Of these three, only the first represents work that can be distributed to other processors. Furthermore, while we expect that disk activity should remain relatively constant (since roughly the same number of LOD scores are always being written to disk, modulo any redundant calculations undertaken to ensure fault tolerance), we might also expect to see network activity increase, given that the root processor must now receive relatively more results from other machines.

**Figure 5:**
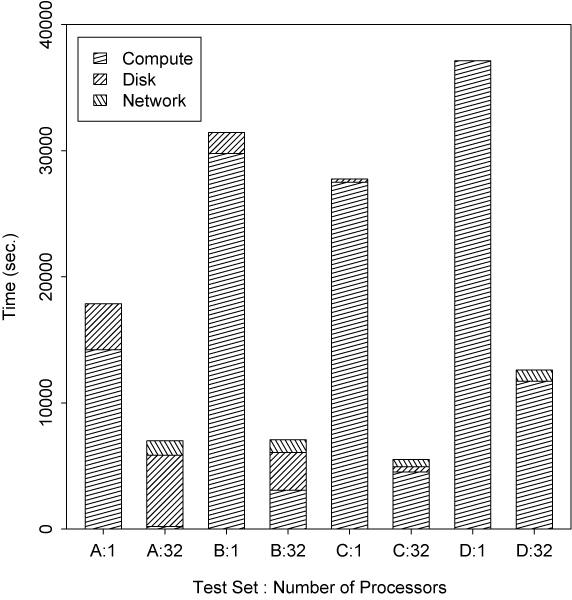
Breakdown of root processor activity.

Figure 5 makes this breakdown explicit for each test set, for the serial and 32 processor case. From the serial computation times, it is clear that set D is the most costly of the four test sets, with potentially the most computation time available for partitioning. However for such sets with fewer but more computationally-intensive calculations (D consists of only four replicated but complex pedigrees), granularity of the available space is a limiting factor and there is a point where the work remaining cannot be partitioned any more finely, forcing some processors to remain idle.

Set A, on the other hand, is the least computationally-intensive of the four sets. However, the grid space for this set is at least twice as large as the space for any other set. As the number of processors grows, the amount of calculation performed by the root processor diminishes to the extent that with n = 32 processors, almost all of the root processor's workload comprises disk and network activities. Test sets B and C represent the most favorable setup here with fairly complex data, and a sufficiently large grid space for distribution.

### Behavior over time

**Figure 6:**
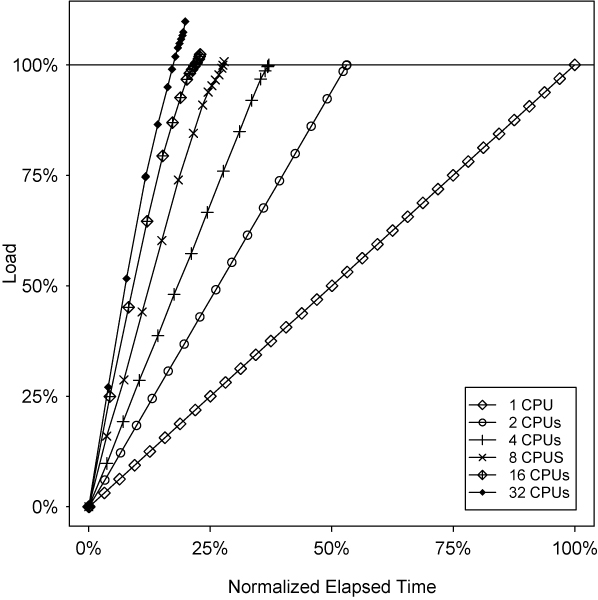
Load completed vs. normalized elapsed time for set C.

We next consider the qualitative behavior of an MLIP calculation over time. Figure 6 plots the *load *or the total number of log-likelihood scores computed as a percentage of the original grid size, versus the elapsed time for data set C, normalized to account for grid size differences.

The single-processor curve shows steady progress through the test, with each regularly-spaced data point corresponding to completion of one of the 32 identical pedigrees in data set C. Since there is only one processor, there is no additional overhead to complicate matters.

The two-processor system also produces a regular, smooth, line, while working its way through its load at about twice the speed. With additional processors, the slope of the lines in the plot continues to increase, although the relative margin of improvement declines. Furthermore, from a qualitative perspective, three striking changes can be seen in the lines generated. First, the spacing of the data points increases and is much less regular than for n = 1 and n = 2. Second, the slope of the line decreases towards the end of the calculation, forming a pronounced "knee" near the end of the trial. Finally, each line generally extends beyond 100% load.

These visual cues tell an interesting story: the increased spacing implies that the root processor is spending relatively more of its time supervising the collection of data from slaves as opposed to making progress on its own calculations. The fact that the spacing is irregular and tends to decrease towards the end of the calculation is because the chunks being handed out to idle slave processors are getting smaller as the remaining workload decreases, leaving less work for the root to complete a pedigree. Moreover, it becomes increasingly difficult to find work for idle processors as the size of the remaining calculation decreases, so some processors are simply told to remain idle. Since the idle slaves are not producing LOD scores, the overall rate at which the load completes is reduced, resulting in a lower slope. Finally, as the number of processors increases, proper load balancing also becomes tricky, leading to an increase in the number of redundant LOD scores computed notwithstanding the fact that the homogeneity of this data (replicated pedigrees) represents a best-case scenario for load balancing.

### Reducing network and disk overhead

The time devoted to network communication and disk access represents a significant obstacle to better performance. Two design changes are evaluated here to help sidestep these obstacles. In the original LIPED implementation, LOD scores are calculated as double precision floating point numbers. Since only 4 to 5 decimal digits of precision are needed for computing the PPL, the cost of disk storage can be halved and the data rate on the network doubled, simply by casting double precision LOD scores to 32-bit floating point.

A second improvement comes from the manner in which these values are written to the disk. The file on the disk is as an ordered repository of LOD scores, indexed by pedigree and parameter space values. Since each processor generally produces LOD scores in sequential order, collections of LOD scores can be efficiently streamed to the disk at the same time, thereby amortizing disk access time and producing higher throughput.

**Figure 7:**
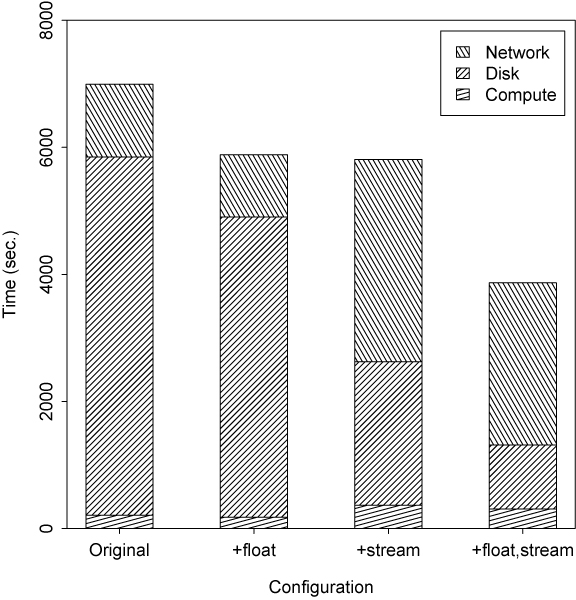
Overhead costs by configuration with 32 processors for set A.

Figure 7 shows the effect of these configuration changes on set A, with a 32 processor hierarchy. Changing representation from double to single precision floating point does indeed reduce both communication costs and the cost of writing the file. Even greater improvements are afforded by streaming data to the disk rather than individually addressing each LOD score as it is written. While the performance for the 32 processor single-precision streaming system for set A is still well short of linear speedup, the final effect on this I/O bound test set is impressive. There is an 80% increase in speedup from 2.55 for the 32 processor double-precision system to 4.59 for the 32 processor single-precision streaming system, a significant improvement.

### Improving performance via better load balancing

One of the hardest problems for any distributed system is how to best keep all available processors equally busy. The granularity issue briefly mentioned previously is one incarnation of this more general problem that arises when the space remaining is not easily apportioned to all available processors. For some datasets (e.g., dataset D) the problem is particularly acute, but it arises to some degree with any dataset, albeit perhaps only at nodes that are nested deeply within the NICE hierarchy. A related problem occurs when the root processor is unable to keep up with the results being generated by all the slave processors in the hierarchy. In this situation, the slaves queue up, lying idle while they wait for service from the root processor. The problem is compounded because any idle slaves wanting additional chunks must also wait in line for service. Finally, inefficiencies arise when the allocation of processors within the hierarchy of processors is suboptimal. Unfortunately, what constitutes the optimal allocation depends on the specifics of the problem being solved, and is not always easy to predict when the data are not as well behaved as our simulated data sets.

Consider, as an illustration of this last point, the following empirical results (see Table 2). In the results reported thus far, all processors were organized in a hierarchy with branching factor limited to 3; that is, no processor has more than 3 child processors. We repeated our test for each of the four datasets with a variety of branching factors, ranging up to a relatively "flat" hierarchy where the root processor had as many as 17 immediate descendent processors. For the most part, a "broader" hierarchy led to reduced speedup. Some of these reductions (e.g., for dataset C) are quite dramatic: using 32 processors in a flat hierarchy led to only an 8% improvement in performance over the single processor case. But for other datasets (e.g., dataset B), peak speedup was obtained when each processor was allowed as many as 9 children.

**Table 2:** Speedups by branching factor (bf)

**bf**	**Set A**	**Set B**	**Set C**	**Set D**
3	4.62	5.07	5.60	3.87
5	4.52	7.39	4.88	2.74
9	4.19	8.76	3.27	2.74
17	3.42	5.03	1.08	2.05

The message here is that load balancing is difficult: no single strategy is likely to be optimal for all possible datasets. Even with the simple, uniform, datasets used here (replicated pedigrees, etc), effective load balancing is likely to rely on a set of heuristics or a collection of adaptive mechanisms that can help fine tune performance while the calculation is underway.

### Performance on real data

**Figure 8:**
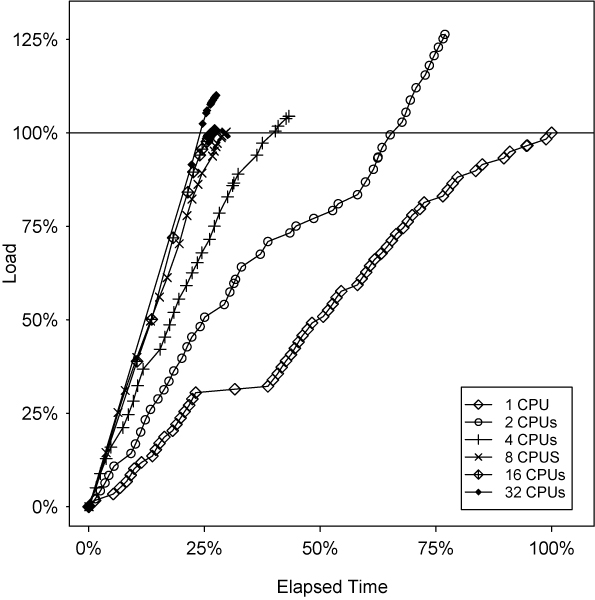
Load completed vs. elapsed time for real data.

In preceding sections the performance of MLIP was evaluated using simulated test sets with homogenous complexity of each grid point. Here, a set of 59 real pedigrees are considered next. These pedigrees consist of 4–14 individuals, genotyped for 34 microsatellite markers, although not all individuals in a pedigree may be genotyped. The grid size (15 × 107) is roughly similar to that used for sets A and B. Figure 8, which shows percent load completed against elapsed time for the real data, can be compared with Figure 6, which plots similar measures for simulated set C.

The most striking observation is that Figure 8 is decidedly less smooth than Figure 6. This fact is to be expected, given that the computation time of an individual pedigree is likely to vary significantly with real data. In particular, one pedigree encountered about 25% of the way through the serial run required about 10% of the overall runtime (this is the plateau in the single processor curve, the central data point on the plateau corresponds to a system checkpoint, not completion of a pedigree). The effect is less noticeable as more processors are added, since the other processors continue to generate LOD scores while the one exceptionally expensive pedigree is being handled.

As with Figure 6, the increased spacing between points with the addition of more processors reflects a change in root processor workload from computation to disk I/O. However, this change is particularly marked here for the 32 processor case. In Figure 8, it can be seen that with 32 processors, the root processor completes the space for its first pedigree only when over 90% of the total LOD scores have already been computed. For both Figures 6 and 8, spacing between successive data points tends to decrease as the trial completes, indicating smaller chunk sizes. Percentage of effort also increases beyond 100% as processors perform redundant calculations in an attempt to ensure fault tolerance. For the real data, however, Figure 8 reveals that the wildly differing costs of each pedigree contribute to broad fluctuations in final percentage of effort.

## Conclusion

This paper described MLIP, a new multiprocessor two-point genetic linkage analysis system that enables statistical calculations based on the full multidimensional parameter space implicit in the linkage likelihood. Empirical results on a broad variety of data have also been provided to support the claim that MLIP does significantly speed up two-point LOD score calculations over the grid space of model parameters necessary for computing the PPL. In keeping with the requirements for the statistic, MLIP not only provides needed flexibility in model parameter specification, but it also stores individual LOD scores, providing users with the ability to create data subsets and change priors during post processing. Obtaining the output in this form provides some additional benefits as well. The likelihood surfaces tend to be irregular with extensive interaction among the parameters. The output produced by MLIP can also be used by visualization software to allow the user to interactively explore the likelihood surface [20-22].

**Figure 9:**
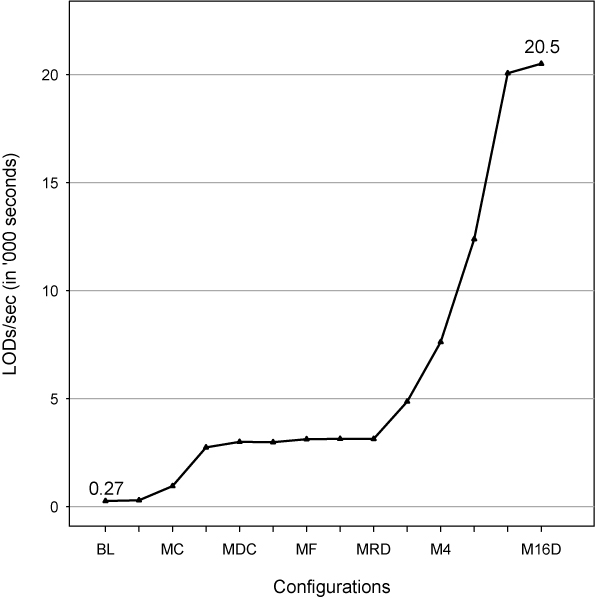
Performance overview.

Figure 9 summarizes the effect of the different improvements on the LOD scores computed per second for a data set comprising 500 large pedigrees with 20 microsatellite markers. The different versions are on the x-axis, while the LOD rate has been plotted along the y-axis. Starting with a baseline rate of approximately 265 using the original LIPED code base; then including changes to strip the code of all I/O; merging the Fortran with the C; allowing for allele downcoding; streaming single-precision output; and finally, using an optimized deep hierarchy instead of a flat one; the rate of output produced and written has gone up to approximately 20,500 LODs/second.

Table 3 puts these results in perspective, summarizing what this means, in practical terms, for the full multidimensional genome scans for which MLIP is already being used. As can be seen, analyses which would have taken two years or more with LIPED can now be routinely performed in days or less by MLIP. Yet, obviously, there is still much room for improvement. In particular, 50 large pedigrees with some unknowns may still take a year or more to analyze.

**Table 3:** From the infeasible to the feasible

	**Large Ped. (500; No Unknowns)**	**Nuclear Ped. (50; Some Unknowns)**	**Large Ped. (50; Some Unknowns)**
LIPED Base	2 years	32 years	59 years
16 CPU MLIP	9 hours	19 days	232 days

The immediate task is to continue making incremental improvements in the load balancing strategy. The current partitioning strategy is not sensitive to predictable differences in expected computation time required for different portions of the grid space. A more fine-grained partitioning strategy would at the very least postpone the granularity problems described earlier. Adaptive partitioning strategies that take cues from observed run times in the course of a calculation are also possible.

While the distributed computing approach adopted here is more coarse-grained, it may be useful to also consider parallelization at the algorithmic level. In such a situation, a hybrid distributed computing infrastructure-allowing both distributed and shared memory-may be a possibility. Use of a grid computing type of infrastructure may perhaps facilitate better time results as opposed to a hierarchy of processors.

Another area of interest is the definition of the grid space itself. Currently, by use of a simple grid specification language, the grid space is defined a priori by the user when the MLIP job is started. Since much of the space might be expected to be relatively "flat," standard adaptive quadrature techniques from numerical integration might be used to allow MLIP to effectively "design its own grid" over the course of an evolving calculation.

Finally, it is a fact that the current core is not fully optimized with respect to the actual likelihood calculation. Well-established differences exist among linkage analysis algorithms. Both these facts suggest that the use of a new computational core, different from LIPED, could significantly extend the capabilities of MLIP. In particular, LIPED is restricted to two-point analyses. Based on what we have learned with MLIP, we are working on constructing a multipoint parallel linkage analysis system [23] based on a reimplementation of the VITESSE [24,25] linkage analysis engine.

As work continues to optimize the performance of the system, the "brute force" grid search method to approximate the PPL as implemented in MLIP, will continue to serve as a benchmark against which future approximations may be judged. Further development and evaluation of fast alternative numerical integration methods is necessarily predicated upon the ability of this program to carry out the calculation based on a full grid-enumeration in an efficient manner.

## Competing interests

The authors declare that they have no competing interests.

## Authors' contributions

MG implemented a large portion of MLIP and was also responsible for all testing of the application and writing its LINUX man page. AMS supervised the implementation and developed the NICE infrastructure utilized by MLIP and further enhanced the infrastructure to provide efficiencies in data streaming and partitioning. VJV conceived of the project and developed the linkage analysis statistic (the PPL) which MLIP was written to compute. All authors contributed equally to the conceptual design of MLIP and in composing this manuscript.
